# Characterization and phylogenetic analysis of the complete mitogenome of sea cucumber *Stichopus ocellatus* (Massin, Zulfigar, Hwai & Boss, 2002) (Aspidochirotida: Stichopodidae)

**DOI:** 10.1080/23802359.2022.2124829

**Published:** 2022-09-28

**Authors:** Shengping Zhong, Longyan Zhao, Guoqiang Huang, Lianghua Huang, Yonghong Liu

**Affiliations:** a Institute of Marine Drugs, Guangxi University of Chinese Medicine, Nanning, China; b Guangxi Engineering Technology Research Center for Marine Aquaculture, Guangxi Institute of Oceanology Co., Ltd., Beihai, China

**Keywords:** Mitochondrial genome, *Stichopus ocellatus*, Stichopodidae, phylogeny

## Abstract

*Stichopus ocellatus*, known as eye-spotted sea cucumber, is a commercially important Stichopodidae holothuroid in Indo-Pacific region for its valuable nutrition and medicinal ingredients. However, because the taxonomic analyses based on morphological characters and molecular data within Aspidochirotida are limited, the deep-level evolutionary relationships of Aspidochirotida are still poorly understood. Here, for providing better insight of future evolutionary and taxonomic classification of Stichopodidae, we report the first complete mitogenome of *S. ocellatus* along with 37 annotated and characterized mitochondrial genes, and the phylogenetic analysis based on mitogenome data reveals sister relationship between *S. ocellatus* and *S. monotuberculatus*.

Stichopodidae, a family of sea cucumbers, is a diverse and conspicuous group of marine benthic ecosystems. Stichopodidae contains more than 128 living species, many of which are ecologically and economically important for enhancing ecosystem nutrient cycling and providing valuable nutrition (Uthicke et al. 2010). Despite being dominant large mobile invertebrates in local ecosystems, the taxonomy of many Stichopodidae species has been revised constantly due to the difficulty in application of traditional taxonomic characters for sea cucumbers (Byrne et al. 2010; Woo et al. 2015; Madduppa et al. 2017). The eye-spotted sea cucumber *Stichopus ocellatus* Massin, Zulfigar, Hwai & Boss, 2002, which inhabits the seabed in the Indo-Pacific region, was known as healthy seafood many years ago but was only described as a validated Stichopodidae species in 2002 (Massin et al. 2002). However, despite its commercial and ecological importance, the genetic diversity and taxonomy of *S. ocellatus* remain poorly explored due to insufficient molecular data and complex taxonomic characteristics, and the mitogenome of *S. ocellatus* has not been sequenced yet. The mitogenome data have proven to be an excellent molecular marker for studying genetic diversity and species identification (Uthicke et al. 2010; Madduppa et al. 2017). Here, we report the complete mitogenome sequence of *S. ocellatus*, which will provide a better understanding of genetic and taxonomic analyses in Stichopodidae.

Tissue samples of one individual *S. ocellatus* were collected from Hainan province, China (Dong Fang, 19.248723 N, 108.484287 E) by local diving fishermen and the whole body specimen (#JP0227) was deposited at Marine Biological Museum, Guangxi Institute of Oceanology, Beihai, China (http://www.gxas.cn/kypt/kxpj/kpcg, Shengping Zhong, shpzhong@foxmail.com). The total genomic DNA was extracted from the muscle of the specimens using an SQ Tissue DNA Kit (OMEGA, Guangzhou, China) following the manufacturer’s protocol. DNA libraries (350 bp insert) were constructed with the TruSeq Nano™ kit (Illumina, San Diego, CA) and were sequenced (2 × 150 bp paired-end) using HiSeq platform at Novogene Company (Beijing, China). Mitogenome assembly was performed by MITObim (Hahn et al. 2013). The complete mitogenome of *S. monotuberculatus* (GenBank accession number: NC_052743) was chosen as the initial reference sequence for MITObim assembly. Gene annotation was performed by MITOS (Bernt et al. 2013).

The complete mitogenome of *S. ocellatus* was 16,260 bp in length (GenBank accession number: NC_062943), and containing the typical set of 13 protein-coding, 22 tRNA, and two rRNA genes, and a putative control region. A total of 37 genes were annotated and 666 nucleotides were identified as putative control region. The overall base composition of the mitogenome was estimated to be A 31.6%, T 29.4%, C 23.8%, and G 15.6%, with a high A + T content of 60.6%, which is similar, but slightly higher than *S. monotuberculatus* (60.4%) within family Stichopodidae (Zhong et al. 2019). The phylogenetic analysis inferred from the concatenated nucleotides sequences of 13 PCGs shows that *S. ocellatus* clustered with *S. monotuberculatus* and *S. horrens* within family Stichopodidae (Figure 1), which is consistent with the phylogenetic analyses of Stichopodidae holothuroids using mitochondrial COI and 16S sequences (Byrne et al. 2010; Madduppa et al. 2017). Our mitogenome data supported the sister relationship between *S. ocellatus*, *S. monotuberculatus*, and *S. horrens*. The complete mitogenome sequence of *S. ocellatus* adds one more mitogenome of family Stichopodidae, which will be useful for better resolving the genetic and taxonomic relationships of Stichopodidae, and related families.

**Figure 1.**
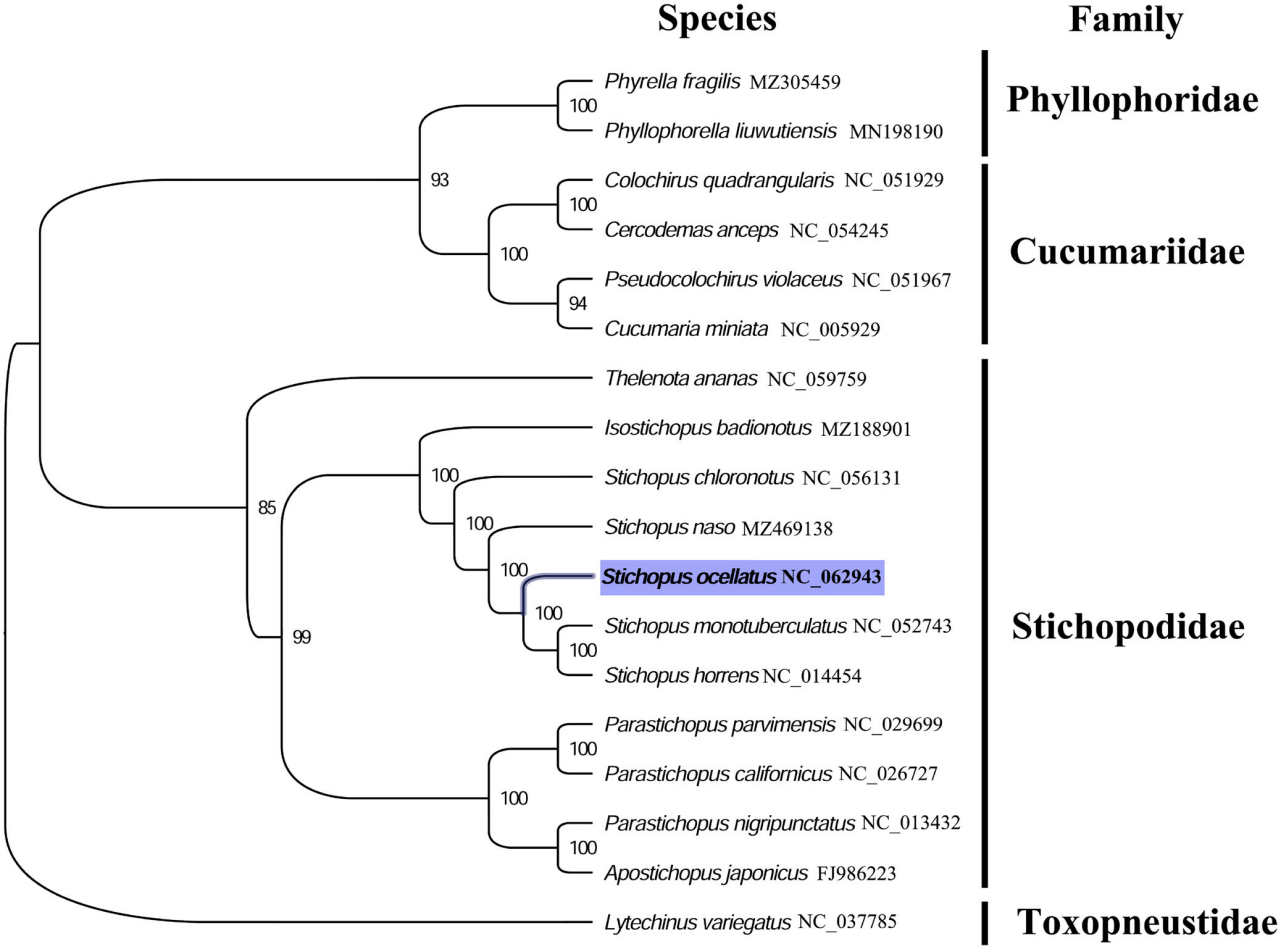
Phylogenetic tree of 18 echinoderm species. The complete mitogenomes were downloaded from GenBank and the phylogenetic tree based on the concatenated nucleotide sequences of 13 mitochondrial PCGs was constructed by maximum-likelihood method via PhyML online server (http://www.atgc-montpellier.fr/phyml/), using GTR substitution model with 100 bootstrap replicates. The bootstrap values are indicated at each branch node, echinoid (*Lytechinus variegatus*) was chosen as the outgroup species.

## Data Availability

The genome sequence data that support the findings of this study are openly available in GenBank of NCBI at https://www.ncbi.nlm.nih.gov/ under the accession no. NC_062943. The associated BioProject, SRA, and Bio-Sample numbers are PRJNA732759, SRR14654595, and SAMN19340382, respectively.
